# A longitudinal approach to understanding boredom during pandemics: The predictive roles of trauma and emotion dysregulation

**DOI:** 10.3389/fpsyg.2022.1050073

**Published:** 2023-01-13

**Authors:** Veerpal Bambrah, Amanda Wyman, John D. Eastwood

**Affiliations:** Boredom Lab, Department of Psychology, York University, Toronto, ON, Canada

**Keywords:** longitudinal, boredom, COVID-19, trauma, emotion dysregulation, emotional clarity, goals

## Abstract

Research during the COVID-19 pandemic and prior outbreaks suggest that boredom is linked to poor compliance with critical lifesaving social distancing and quarantine guidelines, as well as to numerous mental health difficulties. As such, continued understanding on what contributes to boredom is imperative. Extending beyond the roles of constraint, monotony, and trait dispositions (e.g., individual differences in boredom propensity), and informed by prior theories on the emotional contributors of boredom, the current longitudinal study examined the predictive role of “pandemic trauma” on people’s boredom, with a focus on how emotion dysregulation mediates this relationship. Community participants (*N* = 345) completed questionnaires three times across an average of 3 1/2 weeks, rating their pandemic trauma, emotion dysregulation, and boredom over the past week each time. Pandemic trauma was assessed with items querying exposure to coronavirus, as well as the financial, resource-related, and interpersonal pandemic stressors that participants experienced. Emotion dysregulation was assessed with the Difficulties in Emotion Regulation Scale. Boredom was assessed with the short-form Multidimensional State Boredom Scale. The results of a theory-informed mediation model showed that participants’ pandemic trauma at Time 1 positively and modestly predicted their boredom at Time 3 and that this relationship was partially and moderately mediated by participants’ lack of emotional clarity and difficulties with engaging in goal-directed behaviors at Time 2. When people experience pandemic-related trauma, they subsequently struggle to understand their feelings and engage in goal-oriented actions, and, in turn, feel more bored. Theoretical and clinical implications as related to the emotional underpinnings of boredom are discussed.

## 1. Introduction

The coronavirus (COVID-19) outbreak is a global pandemic that began in March 2020. Research suggests that traumatic stress symptoms (e.g., intrusive re-experiencing, heightened arousal) have been particularly prevalent during the pandemic (Cooke et al., 2020; Zhang et al., 2021). Notably, one study by Bridgland et al. (2021) found that participants across the United States, Canada, Australia, New Zealand, and United Kingdom who had come into contact with the coronavirus, lost work/income, had trouble buying supplies, experienced lockdown directives that limited their contact with others, or had experienced changes related to their children/dependents endorsed higher PTSD symptoms than those who did not have these experiences, which suggests that a pandemic can be understood as a traumatic stressor. The COVID-19 pandemic may be the deadliest viral outbreak in more than a century, but emerging research suggests a high and increasingly probability of observing pandemics similar to COVID-19 in the future (e.g., Marani et al., 2021). As such, it is critical to continue to better understand the effects of pandemics, and associated health measures, on people’s emotional wellbeing. The current study examined boredom, the aversive feeling of wanting, but being unable, to engage in satisfying activity (Fahlman et al., 2013), as an index of emotional wellbeing. Previous studies investigating boredom during pandemics have mainly focused on the external constraints and monotony imposed by the pandemic, as well as individual differences in trait dispositions toward boredom, as explanations for this aversive experience. The current study meaningfully extended this prior work, using a longitudinal design to examine the predictive roles of trauma and emotion dysregulation on boredom during the COVID-19 pandemic. Below, we review prior literature on the experience of boredom during pandemics, and then examine the theoretical and empirical literature on the relations of trauma, emotion dysregulation, and boredom.

### 1.1. The experience of boredom during pandemics

Boredom is often triggered by external factors, such as lack of choice, monotony, inappropriate levels of challenge, and devalued activities (Elpidorou, 2018). The feeling can be such a distressing state that people engage in unhealthy behaviors in order to alleviate it (e.g., self-harm, unhealthy eating; Havermans et al., 2015) and seek experiences that elicit a different emotional state—even if that state is negative (Bench and Lench, 2019). Boredom proneness (BP), characterized as the tendency to feel bored more frequently and intensely (Farmer and Sundberg, 1986; Tam et al., 2021), is also related to a host of psychosocial and mental health problems, such as depression, anxiety, substance abuse, addictive behaviors, and risk-taking (see Danckert et al., 2018 for a review).

In the context of a pandemic, people are asked to accept constraints and restrictions on normal behaviors, leaving them with a limited set of available activities that, for many, are monotonous, uninteresting, and void of meaning (Maison et al., 2021). Unsurprisingly, research conducted during the COVID-19 outbreak and the 2003 SARS outbreak suggests that boredom is one of the most commonly experienced feelings (e.g., Reynolds et al., 2008; Barari et al., 2020; Droit-Volet et al., 2020; Li et al., 2021; Martarelli et al., 2021; Martinelli et al., 2021; Wessels et al., 2022), as well as that boredom is a key emotional disincentive to complying with social distancing/quarantine guidelines (e.g., DiGiovanni et al., 2004; Wolff et al., 2020; Bieleke et al., 2021; Boylan et al., 2021; Brosowsky et al., 2021; Drody et al., 2022; but see Westgate et al., 2022 for their emerging findings related to pandemic boredom and risky public health behaviors). Equally important, the feeling of boredom and BP have been linked to various psychological problems among adolescents and adults during the pandemic, such as increased alcohol and substance use (e.g., vaping and smoking), problematic social media and Internet use (i.e., Internet addiction), perceived stress, and psychological distress, including symptoms of depression, anxiety, and insomnia (see Bambrah et al., 2022 for a review). Given the importance of social distancing/quarantine policies in curbing the spread of infectious diseases (e.g., Glogowsky et al., 2021) and the troubling relations between boredom and mental health during COVID-19, it is critical to understand the feeling of boredom as an outcome in its own right.

To date, research (Reynolds et al., 2008; Barari et al., 2020; Droit-Volet et al., 2020; Martarelli et al., 2021; Martinelli et al., 2021; Wessels et al., 2022) has largely underscored constraint, monotony, and lack of challenge imposed by the pandemic, as well as individual differences in BP, as contributors to the feeling of boredom during the COVID-19 and SARS outbreaks. Understandably, the implications of this work highlight integrating adventure, variety, challenge, and excitement in one’s day-to-day life so as to mitigate boredom. For example, colloquial discussion among media outlets suggests various things that people can do and alter within their external environments to “combat” the constraint and monotony associated with boredom (e.g., Ehl, 2021). The current longitudinal study extended this past work by examining two relevant, yet under-studied, predictors of boredom during the COVID-19 outbreak: trauma and emotion dysregulation.

### 1.2. Trauma and boredom

A small body of research has found a positive relationship between trauma and boredom. However, this literature as a whole can be characterized disparate and disconnected, as there is a lack of consistency across studies in how boredom is conceptualized and operationalized. In particular, some studies conceptualize boredom as a problematic, but transient, affective experience (i.e., Stretch et al., 1996a, 1996b; Silove et al., 1997; Pfefferbaum et al., 2007; Schweitzer et al., 2018), whereas other studies conceptualize boredom as a trait disposition (i.e., Chaney and Blalock, 2006; Volkert et al., 2013), but operationalize this with different measures (e.g., Boredom Proneness Scale by Farmer and Sundberg, 1986, vs. the Boredom Susceptibility Scale by Zuckerman, 1979) that are known to be associated with distinct constructs (Mercer-Lynn et al., 2013a, 2013b), thereby highlighting the discrepancy in how trait boredom is conceptualized. Additionally, the types of trauma examined across prior studies are variable, with studies examining boredom among refugee asylum-seekers (Silove et al., 1997; Schweitzer et al., 2018), Gulf War Veterans (Stretch et al., 1996a, 1996b), men with a history childhood abuse (Chaney and Blalock, 2006), adult victims of single or repeated interpersonal trauma (sexual and/or physical trauma; Volkert et al., 2013), and children after the 2001 World Trade Center attacks (Pfefferbaum et al., 2007). Most notably, a clear understanding on *why* there is a positive association between trauma and boredom is lacking from prior work, with some of the studies providing no explanation for the link (i.e., Stretch et al., 1996a, 1996b; Chaney and Blalock, 2006) and other studies providing distinct ideas for the link (e.g., social withdrawal, social exclusion, increased need for stimulation; Silove et al., 1997; Pfefferbaum et al., 2007; Volkert et al., 2013; Schweitzer et al., 2018). Accordingly, the current study not only sought to investigate the link between trauma and boredom as a transient state within the specific context of COVID-19, but also advance our understanding of this link by investigating the mediating role of emotion dysregulation. Our proposal that emotion dysregulation might mediate the relation between trauma and boredom is reinforced by the well-documented literature that suggests that trauma can lead to poor emotion regulation and the emerging understanding that poor emotion regulation might be an internal cause of boredom.

### 1.3. Trauma and emotion dysregulation

With respect to the link between trauma and emotion dysregulation, using Gratz and Roemer’s (2004) integrative conceptualization and corresponding measure, the *Difficulties in Emotion Regulation Scale* (DERS), Ehring and Quack (2010) found that, among a large sample of trauma survivors, PTSD symptom severity was robustly associated with the lack of emotional awareness, lack of emotional clarity, difficulties with engaging in goal-directed behaviors, impulse control difficulties, nonacceptance of emotions, and the belief that there is little that can be done to regulate emotions effectively. Another more recent study (Mekawi et al., 2020) found that people with current PTSD reported more dysregulation across all six DERS subscales, relative to people without PTSD and with lifetime PTSD. This work suggests that people with trauma struggle to attend to, understand, control, and accept their negative emotions, as well as struggle to behave in a manner that is consistent with their goals and lack belief in their ability to effectively regulate their emotions. In other words, when people experience such wide-ranging emotion dysregulation, they often have a limited understanding of what they are feeling and fail to respond accordingly and appropriately.

### 1.4. Emotion dysregulation and boredom

A rationale for why poor emotion regulation might lead to subsequent boredom can be found in psychodynamic and existential theories of boredom. For example, psychodynamic theorists posit that boredom is a side-effect of defensive processes designed to keep verboten desires out of awareness. That is, attempts to keep specific desires out of awareness result in a more diffuse emotional numbing, lack of emotional clarity, and detachment, which, in turn, results in boredom because it is then difficult to determine what one would like to do. Furthermore, attempts to find satisfaction in the external world are never completely satisfying when one’s desires remain unconscious (Fenichel, 1953; Greenson, 1953; Wangh, 1975). Similarly, one strand of existential thought posits that boredom, and its associated lack of emotional clarity, serves as a defense against the overwhelming responsibility of having to continuously actualize oneself through engagement with the world (e.g., White, 1998; Bargdill, 2000). Thus, although different in detail, both psychodynamic and existential theories suggest boredom results when anxiety prevents people from bringing their predicament into focus and prevents people from ‘owning up to’ or ‘putting on the line’ what they desire and who they might become. Because of anxiety of the alternative, people settle for an emotionally numb and disengaged existence and ensuing boredom.

A few correlational studies lend empirical support to the notion that lack of emotional clarity and difficulties with emotion regulation might cause boredom, but, notably, these studies examine the link between dispositional indices of emotion dysregulation and boredom proneness (BP). For example, studies have found that people who possess a restricted capacity for labeling and describing their emotions are more prone to boredom (Seib and Vodanovich, 1998; Gana et al., 2000; Harris, 2000; von Gemmingen et al., 2003; Eastwood et al., 2007). Other work indicates that certain self-regulatory styles that diminish goal pursuit are associated with BP. For example, early work suggests that “state-orientation,” a change-preventing mode of self-regulation, is linked to boredom, specifically finding that BP is positively related to state-oriented “preoccupation” and “hesitation”—the former characterized by intrusive and persevering thoughts associated with unpleasant experiences and the latter characterized by an impaired initiation of an intended action (Blunt and Pychyl, 1998). Other studies have found that BP is positively correlated with the “Assessment” mode of self-regulation, which is characterized by a ruminative evaluative orientation (“do the right thing”) that hampers goal pursuit, and is negatively correlated with the “Locomotion” mode of self-regulation, which is characterized by an action-oriented mode (“just do it”) of goal pursuit (Struk et al., 2015; Mugon et al., 2018).

### 1.5. Current study

It is frequently reported that pandemics (e.g., the SARS outbreak, the COVID-19 pandemic) are associated with increased boredom. Yet, the majority of this empirical and colloquial discussion tends to be descriptive (e.g., documenting the occurrence and intensity of boredom) and prescriptive (e.g., offering ways to cope with boredom) in nature. Moreover, existing work primarily implicates the roles of pandemic-imposed constraint and monotony, and trait dispositions (i.e., BP) in people’s boredom. These prior studies set the stage for a timely and pragmatic research agenda that utilizes rigorous longitudinal methods and analysis to form a novel understanding of boredom by examining other possible contributors, namely, pandemic-related trauma mediated through emotion dysregulation. Driven by the above-reviewed theories of boredom, and by the empirical work on the emotional and self-regulatory correlates of boredom, the current study used a longitudinal design to explore the predictive role of trauma on people’s boredom during COVID-19, with a focus on how emotion dysregulation mediates this relationship. Drawing on the above-reviewed theories, we propose that people respond to pandemic trauma with regulatory strategies characterized by a disconnect from one’s emotional experiences, which, in turn, lead to increased feelings of boredom. Across three time points, we hypothesized that pandemic trauma (at Time 1) would predict greater emotion dysregulation (at Time 2), which, in turn, would predict greater boredom (at Time 3) among people in the general population. Beyond the COVID-19 pandemic, understanding the *internal* (i.e., unrelated to an impoverished *external* environment) and *emotional* contributors of boredom is a critical endeavor, as it may inform the development of interventions for people who chronically struggle with boredom and the wide-ranging mental health problems associated with it.

## 2. Materials and methods

### 2.1. Participants and procedures

Ethics approval was obtained from our academic institution’s research ethics board. All participants were recruited from Qualtrics’ Online Panels, which provide community participants with opportunities to complete online studies. All participants provided informed consent before each data collection time point and Qualtrics compensated participants at each time point with a choice of different gift cards for various American and Canadian retailers. A full description of participant recruitment, including how we determined our sample size, participant attrition, and data exclusions, is available publicly^1^ and is reported elsewhere (Bambrah et al., 2022). Broadly we recruited participants who resided in the United States (U.S.) and Canada. Participants were contacted three times (via Qualtrics’ Online Panels) at the height of the COVID-19 pandemic, between 6 May 2020 and 18 June 2020. At each time point, participants completed a series of questionnaires about their experiences over the past week. At Time 1, participants completed additional measures about their background (e.g., demographic characteristics and pre-existing life stressors). Qualtrics’ Online Panels services mistakenly failed to invite all of the participants who had completed Time 1 to subsequently complete Time 2 questionnaires. Therefore, we recruited additional participants in a second wave to achieve an adequate sample size; collapsing across Wave 1 and Wave 2, the average attrition was 56% from Time 1 to Time 2 and 26% from Time 2 to Time 3. After removing participants who completed questionnaires in an unrealistically short or long time and/or participants who provided similar responses across positively and negatively worded items on questionnaires, the final sample at Time 3 across Waves 1 and 2 consisted of 345 participants (67.83% female), with a mean age of 49.26 years (*SD* = 16.68, range = 18–88). The majority of participants retained within the final sample resided in the U.S. (Wave 1 = 100.00%, Wave 2 = 99.68%).

See Table 1 for the demographic characteristics of the sample. The majority of participants reported living with someone else at the time of the study (*N* = 252, 73.04%); a romantic partner/spouse (61.90%) and children (42.46%) were the most frequently reported cohabitors. The majority of participants were not enrolled in school at the time of the study (*N* = 311, 90.14%). Of those participants who indicated current student enrollment (*N* = 34, 9.86%), more than half were completing courses online (52.94%). The highest level of education for the majority of participants was a high school diploma/GED (*N* = 156, 45.22%). Nearly two-thirds of participants were not currently employed at the time of the study (*N* = 219, 63.48%). Of those participants who indicated current employment (*N* = 126, 36.52%), the majority were working from home (42.06%). The majority of participants reported a yearly household income of $50,000 or less (*N* = 207, 60.00%).

**Table 1:** Sample characteristics (*N* = 345).

	*n*	%		*n*	%
Gender:			Educational background:		
*Male*	110	31.88	*Less than High School Diploma*	16	4.64
*Female*	234	67.83	*High School Diploma/GED*	156	45.22
*Other*	0	0.00	*Some University/College*	72	20.87
*Prefer not to answer*	1	0.29	*Associate’s Degree*	23	6.67
			*Bachelor’s Degree/Diploma*	54	15.65
Country of residence:			*Graduate Degree (MA, PhD)*	24	6.96
*United States*	344	99.71			
*Canada*	1	0.29	Current employment:	126	36.52
			*Not working at this time*	36	28.57
Living with someone:	252	73.04	*Working from home*	53	42.06
*Romantic partner/spouse*	156	61.90	*Working outside of home*	37	29.37
*Parents*	51	20.24			
*Siblings*	30	11.90	Household yearly income:		
*Children*	107	42.46	*Less than $25,000*	104	30.14
*Grandparents*	3	1.19	*$25,000–$50,000*	103	29.86
*Friends*	15	5.95	*$50,001–$75,000*	70	20.29
*Pets*	41	16.27	*$75,001–$100,000*	34	9.86
*Other*	14	5.56	*Greater than $100,000*	34	9.86
					
Current student enrollment:	34	9.86	Pre-pandemic stress-type:		
*Education is on pause*	14	41.18	*Divorce/separation*	25	7.25
*Completing courses online*	18	52.94	*Family conflicts*	83	24.06
*Completing courses in-person*	2	5.88	*Conflicts in work life*	28	8.12
			*Conflicts with neighbors*	26	7.54
Pre-pandemic stress—#:			*Illness of a loved one*	62	17.97
*0*	22	6.38	*Death of a loved one*	84	24.35
*1*	115	33.33	*Adjustment due to retirement*	21	6.09
*2*	89	25.80	*Unemployment*	68	19.71
*3*	56	16.23	*Too much/too little work*	53	15.36
*4*	31	8.99	*Pressure to meet deadlines*	27	7.83
*5*	11	3.19	*Moving to a new home*	55	15.94
*6*	9	2.61	*Financial problems*	124	35.94
*7*	4	1.16	*Own serious illness*	42	12.17
*8*	2	0.58	*Serious accident*	9	2.61
*9*	5	1.45	*Assault*	10	2.90
*10*	1	0.29	*Termination of leisure activity*	13	3.77
			*Other*	63	18.26
	*M*	*SD*			
Age	49.26	16.68			

Our sample included participants from 47 states in the U.S. The most highly represented states, specifically consisting of 20 or more participants, were Florida (9.01% of the U.S. sample), California (8.14%), New York (7.27%), and Texas (6.69%). Confirmed COVID-19 cases in the U.S. rose from 1.2 million on 6 May 2020 to 2.2 million on 18 June 2020, an increase of 1 million cases during this period of data collection (Johns Hopkins University Coronavirus Research Center, 2020). The total number of deaths related to COVID-19 rose from 76.9 thousand on 6 May 2020 to 121.1 thousand on 18 June 2020, an increase of 44.2 thousand deaths (Johns Hopkins University Coronavirus Research Center, 2020). In the month prior to the study (April 2020), the unemployment rate in the U.S. increased by 10.3% points to 14.7%—the highest rate and the largest over-the-month increase in the history of the data collected by the U.S. Bureau of Labor Statistics (2020a). Unemployment rose sharply among all major worker groups. In May 2020, the unemployment rate declined slightly to 13.3%, reflecting a limited resumption of economic activity that was curtailed in March and April due to efforts to contain the pandemic (U.S. Bureau of Labor Statistics, 2020b).

### 2.2. Measures

Participants completed a large number of questionnaires of which a subset of responses are reported here. See Table 2 for the coefficient omega estimates and descriptive statistics (range, mean, standard deviation) of the measures that were administered at Times 1, 2, and 3 of the current study.

**Table 2:** Coefficient omega estimates and descriptive statistics for continuous measured variables at Times 1, 2, and 3 (*N* = 345).

Variable:		Time 1:			Time 2:			Time 3:		
	Range	ω	*M*	*SD*	ω	*M*	*SD*	ω	*M*	*SD*
**Pandemic trauma:**										
Exposure	4.00–28.00	0.91	5.97	4.84	0.92	6.23	5.24	0.92	6.15	5.08
Financial	2.00–14.00	0.76	5.71	3.97	0.76	5.59	3.88	0.82	5.32	3.94
Resource	2.00–14.00	0.92	6.57	4.16	0.91	6.64	4.15	0.93	6.16	4.05
Interpersonal	4.00–28.00	0.75	13.43	6.70	0.79	13.60	6.82	0.78	12.74	6.74
**DERS-18:**										
Clarity	3.00–15.00	0.89	5.70	3.24	0.87	5.66	3.08	0.90	5.50	3.14
Goals	3.00–15.00	0.89	6.86	3.52	0.92	6.60	3.64	0.92	6.37	3.51
Impulse	3.00–15.00	0.92	5.24	3.22	0.92	5.10	3.21	0.92	4.91	3.03
Nonacceptance	3.00–15.00	0.90	5.86	3.36	0.89	5.81	3.38	0.90	5.45	3.21
Strategies	3.00–15.00	0.87	5.82	3.30	0.90	5.76	3.43	0.89	5.41	3.15

Boredom	6.00–42.00	0.87	21.74	9.13	0.89	21.68	9.51	0.89	21.29	9.56

#### 2.2.1. Time 1

##### 2.2.1.1. Demographics

Participants reported their age, gender, country of residence (U.S. or Canada), living arrangement, educational background and enrollment status, employment status, and yearly household income (see Table 1 for more details).

##### 2.2.1.2. Pre-existing stressors

Participants responded to one question that queried the number of burdensome life stressors—preceding the coronavirus pandemic—that they had experienced. Drawn from the Adjustment Disorder–New Module 20 Questionnaire (ADNM-20; Einsle et al., 2010), participants were provided a list of 16 stressful life events (e.g., assault) and they were asked to indicate which event(s), if any, happened to them during the past 2 years before the pandemic and remains a “very strong” burden to them/has burdened them in the past 6 months. Participants were also able to report stressful experiences that were not included on the list. A higher total score indicated a greater number of burdensome pre-existing (pre-pandemic) stressors. As shown in Table 1, the greatest proportion of participants (*N* = 115, 33.33%) reported at least one life stressor that preceded the COVID-19 pandemic and remains a burden for them. Financial problems (*N* = 124, 35.94%), death of a loved one (*N* = 84, 24.35%), and family conflicts (*N* = 83, 24.06%) were the three most frequently reported burdensome pre-existing stressors in the sample.

#### 2.2.2. Times 1 to 3

##### 2.2.2.1. Pandemic trauma

Drawing on the above-described findings from Bridgland et al. (2021), we assessed participants’ *pandemic trauma* with four indicator variables: (1) *exposure to coronavirus* (e.g., personal coronavirus diagnosis/symptoms and proximity to others with a coronavirus diagnosis/symptoms), as well as the (2) *financial* (e.g., losing job-related income), (3) *resource-related* (e.g., difficulty getting necessary supplies), and (4) *interpersonal* (e.g., difficulty socially connecting) stressors of the pandemic. The first three variables were measured using subscales from the *Coronavirus Experiences Questionnaire* and *Coronavirus Impacts Questionnaire* developed by Conway et al. (2020) and the fourth variable was measured using four items created by the authors of the current study. A total of 12 items were rated using a 7-point scale (1 = *not true of me at all* to 7 = *very true of me*). The exposure, financial, resource-related, and interpersonal subscales had acceptable-to-excellent internal reliability across all three time points (see Table 2 for the coefficient omega estimates of all measures administered between Times 1 and 3).

##### 2.2.2.2. Emotion dysregulation

The 18-item DERS (DERS-18) is composed of the items that load most strongly onto their respective subscale from the original measure (i.e., all factor loadings > 0.75; Victor and Klonsky, 2016) and was used to assess participants’ emotion dysregulation over the past week: (1) *clarity* (i.e., not knowing the emotions that one is experiencing); (2) *goals* (i.e., difficulties concentrating and accomplishing goals/tasks when experiencing negative emotions); (3) *impulse* (i.e., difficulties remaining in control of one’s behavior when experiencing negative emotions); (4) *nonacceptance* (i.e., having negative secondary emotional responses to one’s negative emotions or nonaccepting reactions to one’s distress); and (5) *strategies* (i.e., believing that there is little that can be done to regulate one’s emotions effectively once upset). The *awareness* subscale was excluded from the current study, as emerging research shows that a revised five-factor model that excludes the subscale provides a better fit to the data and that the subscale possesses low associations with the other DERS subscales (e.g., Hallion et al., 2018 for a review). Participants rated each item using a 5-point scale [1 = *almost never (0%–10%) to 5 = almost always (91%–100%)*], with a higher subscale score indicating greater difficulties with that facet of emotion regulation over the past week. The subscales had good-to-excellent internal consistency across all three time points.

##### 2.2.2.3. Boredom

The short-form Multidimensional State Boredom Scale (MSBS) assessed participants’ boredom over the past week. From the 28-item MSBS (Fahlman et al., 2013), participants rated six items using a 7-point scale (1 = *strongly disagree* to 7 = *strongly agree*), with a higher total score indicating greater boredom over the past week. A series of confirmatory factor analyses (CFA) conducted with undergraduate and community samples from other studies suggests that a one-factor structure of the six-item MSBS fits the data well and has good internal consistency: CFI range = 0.956 to 0.989, RMSEA range = 0.052 to 0.086, SRMR range = 0.025 to 0.045, and ω range = 0.79 to 0.85 (e.g., Eastwood and Bambrah, 2021). A CFA confirmed that a one-factor structure of the six-item MSBS had an acceptable-to-good fit with the data of the current study across all three time points: CFI range = 0.961 to 0.981, RMSEA range = 0.079 to 0.107, and SRMR range = 0.028 to 0.042. The six-item MSBS also had good internal reliability across all time points.

### 2.3. Data analysis

Data were analyzed using R (version 4.2.1; R Core Team, 2022), with the packages *car* (version 3.1–0; Fox and Weisberg, 2019), *ggplot2* (version 3.3.6; Wickham, 2016), *lavaan* (version 0.6–12; Rosseel, 2012), *boot* (version 1.3–28; Canty and Ripley, 2021), and *MBESS* (version 4.9.1; Kelley, 2007).

#### 2.3.1. Mediation analyses

**Figure 1:**
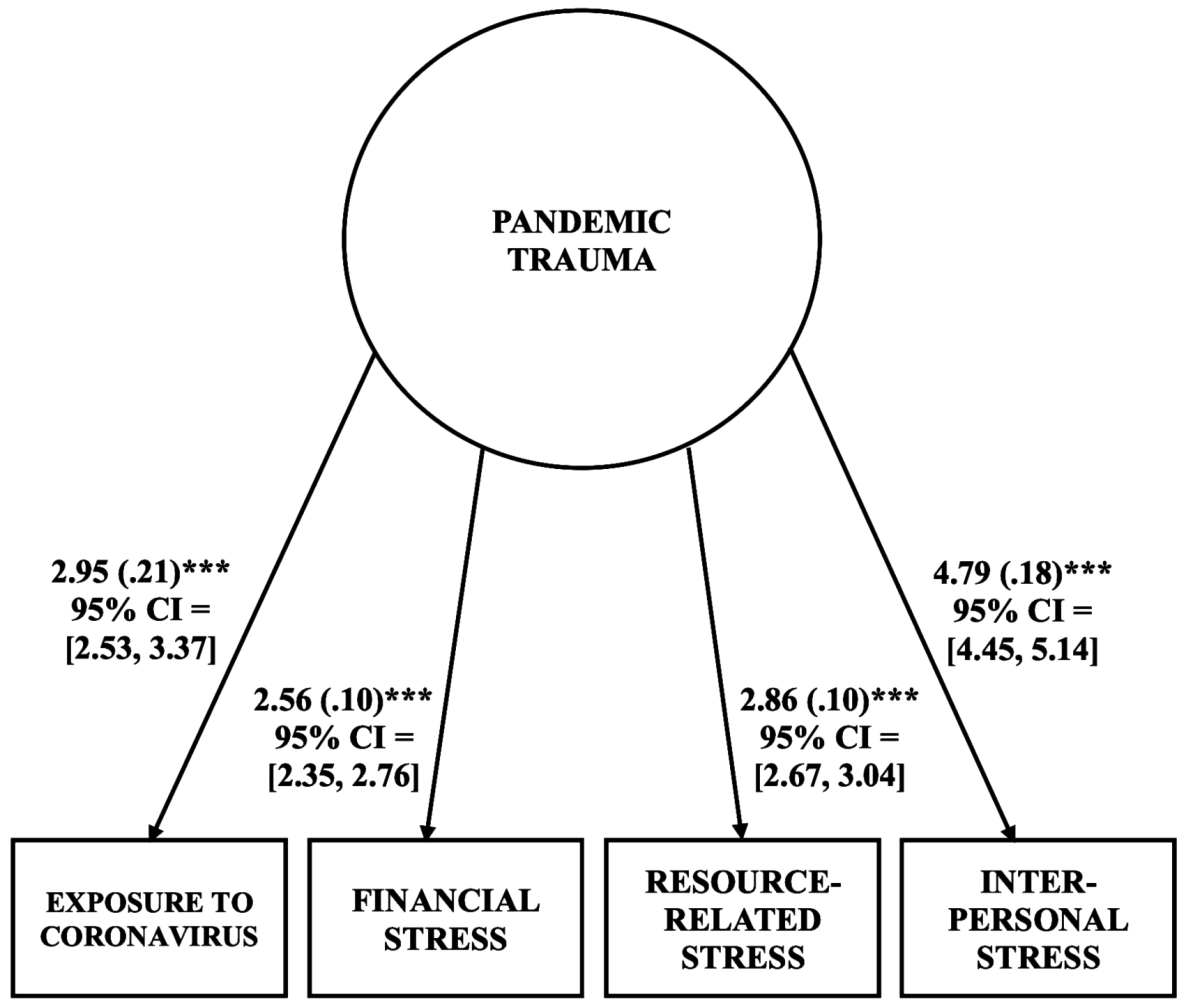
Model of pandemic trauma latent variable (at Time 1, *N* = 1,356). Unstandardized estimates are shown with standard errors in parentheses and with the 95% bias-corrected CI in brackets. The latent variable accounted for 27.90%, 38.10%, 51.40%, and 49.70% of the variable in the exposure to coronavirus, financial stress, resource-related stress, and interpersonal stress indicators, respectively. All *p*’s < 0.001.

**Figure 2:**
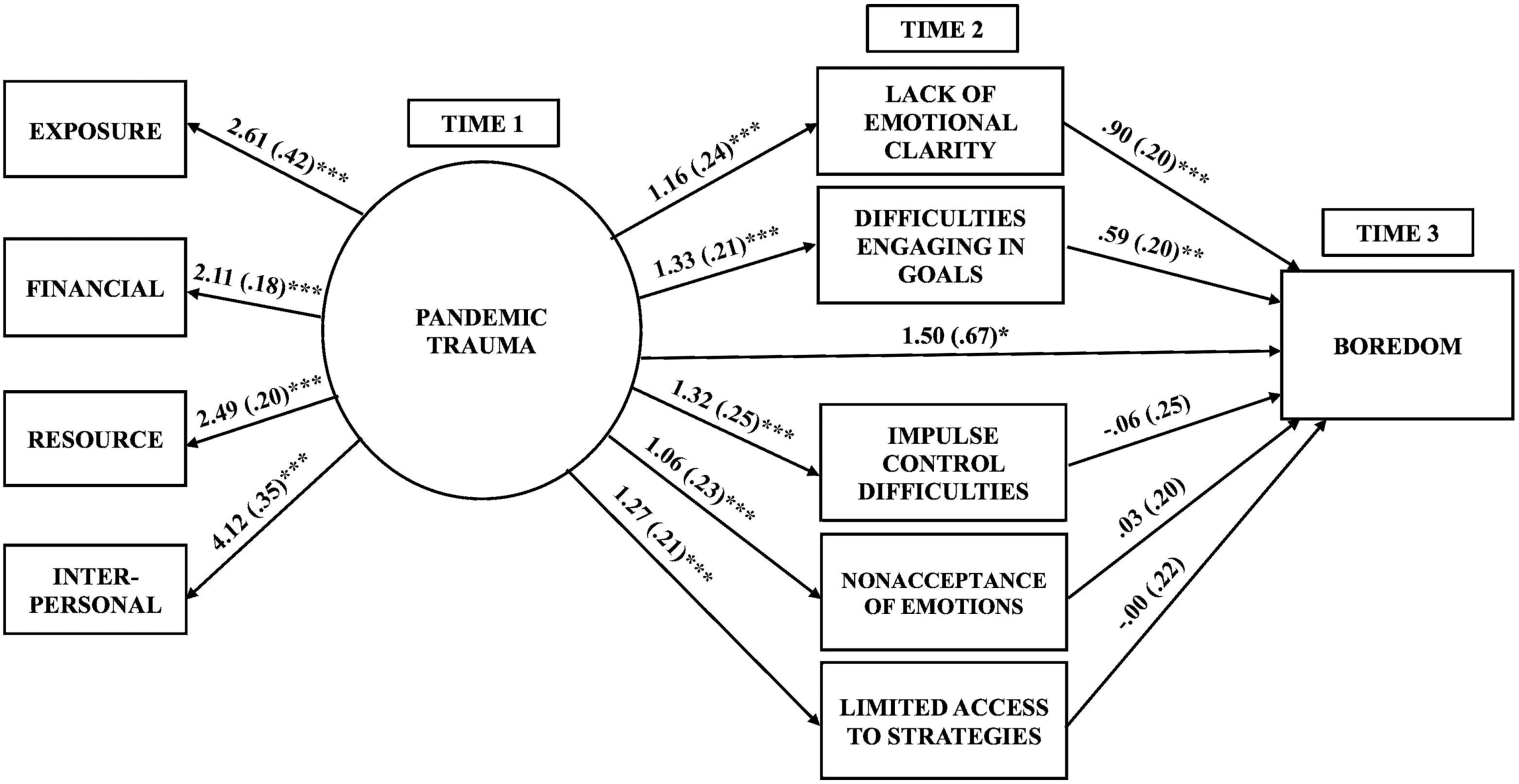
Hypothesized mediation model predicting boredom (*N* = 344). Hypothesized mediation model (*N* = 344) of the pathways to boredom during the coronavirus pandemic. Unstandardized estimates with standard errors in parentheses are shown for all of the paths. Pre-pandemic stress, age, and gender were included as covariates, but are not depicted in the figure (see “Results” for findings related to these covariates). All five mediators at Time 2 were set to covary with each other, but this is not depicted in the model. ^***^p < 0.001; ^**^p < 0.01; ^*^p < 0.05.

To examine our hypotheses, we specified a structural equation model with one latent variable and seven observed variables using the *lavaan* package (Rosseel, 2012). The independent variable in the model was the latent variable of *pandemic trauma* at Time 1, as assessed by participants’ exposure to coronavirus, as well as the financial, resource-related, and interpersonal stressors of the pandemic. We conducted a confirmatory factor analysis (CFA) to verify that a one-factor structure of the latent variable fit the data well; these results are presented in Figure 1. The mediator variables in the model were participants’ emotion dysregulation at Time 2, as measured by the five subscales of the DERS-18 (clarity, goals, impulse, nonacceptance, and strategies); these five subscales were set to covary in the model. The dependent variable in the model was boredom at Time 3, as measured by the total score of the short-form MSBS. Participants’ pre-existing stress was included as a covariate. As it is plausible that participants with more burdensome stressors prior to the pandemic would experience greater pandemic trauma, greater emotion dysregulation, and greater boredom, the *pandemic trauma* latent variable (at Time 1), the DERS-18 subscale scores (at Time 2), and the short-form MSBS total score (at Time 3) were regressed on to this covariate. These variables were also regressed on to age and gender, which were included as covariates. The hypothesized mediation model is depicted in Figure 2.^2^

The robust maximum likelihood estimator (MLR) was used to accommodate violations of the normality assumption. We used the following criteria as an indication of good model fit: comparative fit index (CFI) > 0.95; root mean square error of approximation (RMSEA) < 0.06; and standardized root mean square residual (SRMR) < 0.08 (Hu and Bentler, 1999; as cited in Lai and Green, 2016); CFI values between 0.90 and 0.95 and RMSEA values between 0.05 and 0.10 suggest “acceptable” fit (Browne and Cudeck, 1992; MacCallum et al., 1996; McDonald and Ho, 2002; as cited in Lai and Green, 2016). If the model did not fit the data well, we planned to examine modification indices for potential sources of misfit and respecify the model. Given the post-hoc nature of this process, we planned to only respecify the model based on modification indices > 10 that made conceptual sense within our theoretical framework. Once the final mediation model was specified, bootstrapping (with 5,000 samples) was used to estimate the standard errors and 95% bias-corrected confidence intervals (CIs) for all unstandardized path coefficients (a, b, c’, and indirect) in the mediation model. Following recommendations by Steinberg and Thissen (2006) and Pek and Flora (2018) on the reporting of effect sizes in original psychological research, including structural equation models, we present and discuss the unstandardized parameter estimates within the mediation model.

## 3. Results

### 3.1. Descriptive analyses

A series of Independent *t*-tests found no statistically significant differences between participants who were recruited in Wave 1 (*n* = 36) and participants who were recruited in Wave 2 (*n* = 309) on the four pandemic trauma indicator variables at Time 1; the five DERS-18 subscale scores at Time 2; the short-form MSBS total score across at Time 3; age; and the number of burdensome pre-existing (pre-pandemic) stressors reported (all *p*’s > 0.05). A Chi-square test also found no statistically significant difference between groups on gender (i.e., 1 = male, 2 = female; the one participant who did not report their gender was excluded from data analyses). Thus, all retained participants at Time 3 were analyzed together. Histograms and boxplots showed that the continuous variables in the hypothesized mediation model reasonably approximated normal distributions. Bivariate scatterplots showed linear relationships between all variables specified in the mediation model (i.e., the four indicators of pandemic trauma, the five facets of emotion dysregulation, boredom, and the covariates).

### 3.2. Mediation analyses

#### 3.2.1. Pandemic trauma latent variable

Applying a CFA to scores for (a) exposure to coronavirus, (b) financial, (c) resource-related, and (d) interpersonal stressors of the pandemic, we created a latent variable of *pandemic trauma*, which served as the independent variable for the hypothesized mediation model. See Figure 1 for the unstandardized factor loadings and corresponding 95% bias-corrected CIs. As expected, a one-factor structure of these four indicators (among retained participants who completed Time 1, *N* = 1,356) fit the data well: χ^2^(2) = 9.929, *p* = 0.007, CFI = 0.994, RMSEA = 0.051, and SRMR = 0.016. The latent variable accounted for between 27.90 and 51.40% of the variance in the indicators and it accounted for significant variance in all of the indicators (all *p*’s < 0.001 for the factor loadings).

#### 3.2.2. Hypothesized mediation model

Table 3 presents the zero-order correlations between all key measured variables in the mediation model. The hypothesized mediation model (*N* = 344; one participant who did not report their gender was excluded from this analysis) demonstrated an acceptable-to-good fit with the data, χ^2^ (30) = 97.728, *p* < 0.001, CFI = 0.966, RMSEA = 0.083, and SRMR = 0.043, and it was used as the final model as no modification indices met our criteria for consideration. The model accounted for 46.00% of the variance in boredom at Time 3.

**Table 3:** Zero-order correlations between indicator, mediator, and outcome variables in hypothesized mediation model (*N* = 345).

	1.	2.	3.	4.	5.	6.	7.	8.	9.	10.
1.	-									
2.	0.40^***^	-								
3.	0.35^***^	0.46^***^	-							
4.	0.42^***^	0.38^***^	0.54^***^	-						
5.	0.44^***^	0.26^***^	0.30^***^	0.36^***^	-					
6.	0.35^***^	0.31^***^	0.37^***^	0.37^***^	0.66^***^	-				
7.	0.45^***^	0.27^***^	0.35^***^	0.36^***^	0.69^***^	0.75^***^	-			
8.	0.40^***^	0.30^***^	0.30^***^	0.30^***^	0.62^***^	0.73^***^	0.71^***^	-		
9.	0.39^***^	0.27^***^	0.38^***^	0.35^***^	0.68^***^	0.83^***^	0.79^***^	0.74^***^	-	
10.	0.35^***^	0.26^***^	0.31^***^	0.42^***^	0.58^***^	0.58^***^	0.51^***^	0.50^***^	0.54^***^	-

Variable Names: 1. Exposure to coronavirus (Time 1), 2. Financial stress (Time 1), 3. Resource-related stress (Time 1), 4. Interpersonal stress (Time 1), 5. Lack of emotional clarity (Time 2), 6. Difficulties with engaging in goal-directed behaviors (Time 2), 7. Impulse control difficulties (Time 2), 8. Nonacceptance of emotions (Time 2), 9. Limited access to emotion regulation strategies (Time 2), 10. Boredom (Time 3).

*** *p *< 0.001.

##### 3.2.2.1. Direct paths

Unstandardized path coefficients for the hypothesized model are shown in Figure 2. The total effect of pandemic trauma at Time 1 on boredom at Time 3 (path *c*; not depicted in Figure 2) was statistically significant and positive (*B* = 3.26, *SE* = 0.59, *p* < 0.001, 95% CI = [2.10, 4.43], *β* = 0.39), while statistically controlling for participants’ pre-existing stress, age, and gender. The direct effect of pandemic trauma at Time 1 on boredom at Time 3 (path *c’*; depicted in Figure 2) reduced but remained statistically significant and positive when the mediators were additionally included in the hypothesized model (*B* = 1.50, *SE* = 0.67, *p* = 0.025, 95% CI = [0.26, 2.89], *β* = 0.18). An increase in pandemic trauma at Time 1 uniquely predicted a 1.50-point increase in boredom at Time 3, as measured by the MSBS, over and above participants’ pre-existing stress, age, gender, and emotion dysregulation at Time 2.

As expected, participants’ pandemic trauma at Time 1 significantly predicted their lack of emotional clarity (*B* = 1.16, *SE* = 0.24, *p* < 0.001, 95% CI = [0.68, 1.63], *β* = 0.43), difficulties with engaging in goal-directed behaviors (*B* = 1.33, *SE* = 0.21, *p* < 0.001, 95% CI = [0.92, 1.73], *β* = 0.42), difficulties with impulse control (*B* = 1.32, *SE* = 0.25, *p* < 0.001, 95% CI = [0.84, 1.81], *β* = 0.47), nonacceptance of negative emotions (*B* = 1.06, *SE* = 0.23, *p* < 0.001, 95% CI = [0.63, 1.51], *β* = 0.36), and limited access to emotion regulation strategies (*B* = 1.27, *SE* = 0.21, *p* < 0.001, 95% CI = [0.86, 1.67], *β* = 0.42) at Time 2 (path *a*), while controlling for their pre-existing stress age, and gender. In other words, an increase in pandemic trauma at Time 1 uniquely predicted more than a 1.00-point increase on the DERS-18, across multiple facets of participants’ emotion dysregulation at Time 2. In turn, poor clarity of emotions at Time 2 (*B* = 0.90, *SE* = 0.20, *p* < 0.001, 95% CI = [0.50, 1.29], *β* = 0.29) and difficulties with concentrating and accomplishing goals when experiencing negative emotions at Time 2 (*B* = 0.59, *SE* = 0.20, *p* = 0.004, 95% CI = [0.20, 1.00], *β* = 0.23) significantly and positively predicted participants’ boredom at Time 3 (path *b*), over and above their pre-existing stress, age, gender, pandemic trauma at Time 1, and the other facets of emotion dysregulation at Time 2. A one-point increase in these two facets of emotion dysregulation at Time 2, as measured by the DERS-18, uniquely predicted respective increases of 0.90 and 0.59 points in boredom at Time 3, as measured by the MSBS. The effects of impulse control difficulties (*B* = −0.06, *SE* = 0.25, *p* = 0.800, 95% CI = [−0.56, 0.44], *β* = −0.02), nonacceptance of negative emotions (*B* = 0.03, *SE* = 0.20, *p* = 0.880, 95% CI = [−0.35, 0.44], *β* = 0.01), and limited access to emotion regulation strategies (*B* = −0.00, *SE* = 0.22, *p* = 0.990, 95% CI = [−0.39, 0.45], *β* = −0.00) at Time 2 on boredom at Time 3 were small and non-significant.

Though not depicted in Figure 2, participants’ pre-existing stress was positively related to their pandemic trauma at Time 1 (*B* = 0.22, *SE* = 0.05, *p* < 0.001, 95% CI = [0.13, 0.31], *β* = 0.34), difficulties with concentrating and accomplishing goals at Time 2 (*B* = 0.22, *SE* = 0.11, *p* = 0.045, 95% CI = [−0.01, 0.42], *β* = 0.11), nonacceptance of negative emotions at Time 2 (*B* = 0.30, *SE* = 0.11, *p* = 0.009, 95% CI = [0.06, 0.51], *β* = 0.16), and boredom at Time 3 (*B* = 0.69, *SE* = 0.30, *p* = 0.021, 95% CI = [0.10, 1.26], *β* = 0.13). Older participants (*B* = −0.02, *SE* = 0.00, *p* < 0.001, 95% CI = [−0.03, −0.01], *β* = −0.26) and female participants (*B* = −0.04, *SE* = 0.02, *p* = 0.039, 95% CI = [−0.07, −0.00], *β* = −0.14) reported less pandemic trauma at Time 1. Older participants also endorsed less emotion dysregulation at Time 2, specifically less difficulties with emotional clarity (*B* = −0.03, *SE* = 0.01, *p* = 0.006, 95% CI = [−0.05, −0.01], *β* = −0.15), concentrating and accomplishing their goals (*B* = −0.05, *SE* = 0.01, *p* < 0.001, 95% CI = [−0.07, −0.03], *β* = −0.23), controlling their impulses (*B* = −0.04, *SE* = 0.01, *p* < 0.001, 95% CI = [−0.05, −0.02], *β* = −0.18), accepting their negative emotions (*B* = −0.03, *SE* = 0.01, *p* < 0.001, 95% CI = [−0.05, −0.02], *β* = −0.17), and the belief in their emotion regulation abilities (*B* = −0.05, *SE* = 0.01, *p* < 0.001, 95% CI = [−0.07, −0.03], *β* = −0.24). Older participants additionally reported fewer burdensome pre-existing stressors (*B* = −3.83, *SE* = 1.42, *p* = 0.007, 95% CI = [−6.64, −0.99], *β* = −0.13). Finally, age (*B* = −0.05, *SE* = 0.03, *p* = 0.063, 95% CI = [−0.10, 0.00], *β* = −0.09) and gender (*B* = −0.06, *SE* = 0.09, *p* = 0.497, 95% CI = [−0.23, 0.11], *β* = −0.03) were unrelated to boredom at Time 3.

##### 3.2.2.2. Indirect paths

Unstandardized estimates and the 95% CIs for the indirect paths are shown in Table 4. Pandemic trauma at Time 1 predicted an increase of 1.04 points in boredom at Time 3 (*SE* = 0.30, *p* < 0.001, 95% CI = [0.57, 1.76], *β* = 0.13) through its increased effects on participants’ lack of emotional clarity at Time 2. Similarly, pandemic trauma at Time 1 predicted an increase of 0.79 points in boredom at Time 3 (*SE* = 0.29, *p* = 0.006, 95% CI = [0.30, 1.46], *β* = 0.09) through its increased effects on participants’ difficulties with engaging in goal-directed behaviors at Time 2. None of the other indirect pathways were supported (Table 4). The total indirect effect was also significant (*B* = 1.78, *SE* = 0.37, *p* < 0.001, 95% CI = [1.16, 2.65], *β* = 0.21). Examination of the standardized indirect effects indicated medium effect sizes for the specific indirect effects of emotional clarity and goals, as well as a large effect size for the total indirect effect (see Table 4).

**Table 4:** Unstandardized and standardized estimates and confidence intervals for indirect pathways to boredom (*N* = 344).

Indirect pathway	Unstandardized estimate: *B (SE)*	95% CI	Standardized estimate: *β*
Pandemic trauma ➔ lack of emotional clarity ➔ boredom	1.04 (0.30)^***^	[0.57, 1.76]	0.13
Pandemic trauma ➔ difficulties engaging in goals ➔ boredom	0.79 (0.29)^**^	[0.30, 1.46]	0.09
Pandemic trauma ➔ impulse control difficulties ➔ boredom	−0.08 (0.34)	[−0.80, 0.55]	−0.01
Pandemic trauma ➔ nonacceptance of emotions ➔ boredom	0.03 (0.21)	[−0.39, 0.47]	0.00
Pandemic trauma ➔ limited regulation strategies ➔ boredom	−0.00 (0.27)	[−0.53, 0.55]	0.00
Pandemic trauma ➔ sum of indirect effects ➔ boredom	1.78 (0.37)^***^	[1.16, 2.65]	0.21

An indirect path between Pandemic Trauma and Boredom is one wherein the 95% CI does not contain zero. The bias-corrected 95% CIs are based on 5,000 bootstrapped samples.

*** *p* < 0.001;

** *p* < 0.01;

^*^p < 0.05.

### 3.3. Exploratory analyses

The link between boredom proneness (BP) and the feeling of boredom has been well-established both prior to and during the pandemic (e.g., Mercer-Lynn et al., 2014; Martarelli et al., 2021), underscoring the relevance of BP to people’s experience of boredom. We estimated a post-hoc mediation model^3^, which would allow us to explore the predictive relevance of people’s pandemic trauma and emotion dysregulation on their boredom during the pandemic, while accounting for their BP. Participants’ BP was assessed at Time 1 using the six-item Trait Boredom Scale (Gorelik and Eastwood, 2019 under review, ω = 0.90). Supported by the BP literature reviewed in the introduction, paths from BP to boredom (at Time 3) and to emotion dysregulation (at Time 2) were added in the exploratory mediation model. Additionally, BP was set to covary with participants’ pre-existing stress, age, and gender. The exploratory mediation model demonstrated an acceptable-to-good fit with the data, χ^2^(34) = 136.924, *p* < 0.001, CFI = 0.953, RMSEA = 0.096, and SRMR = 0.072. The results for the *a*, *b*, and indirect paths, which are presented in Table 5, are consistent with the results observed in our hypothesized mediation model. The total effect of pandemic trauma at Time 1 on boredom at Time 3 (path *c*) was statistically significant and positive (*B* = 1.59, *SE* = 0.55, *p* = 0.004, 95% CI = [0.52, 2.64], *β* = 0.19), while controlling for participants’ BP, pre-existing stress, age, and gender. The direct effect of pandemic trauma at Time 1 on boredom at Time 3 (path *c’*) was no longer statistically significant when emotion dysregulation at Time 2, as measured by the five DERS subscales, was added to the model (*B* = 0.57, *SE* = 0.58, *p* = 0.320, 95% CI = [−0.50, 1.75], *β* = 0.07). Most notably, however, even when BP was included in the model, participants’ lack of emotional clarity (*B* = 0.56, *SE* = 0.23, *p* = 0.016, 95% CI = [0.22, 1.17], *β* = 0.07) and difficulties with engaging in goal-directed behaviors (*B* = 0.46, *SE* = 0.22, *p* = 0.034, 95% CI = [0.11, 0.98], *β* = 0.06) at Time 2 fully mediated the relationship between pandemic trauma at Time 1 and boredom at Time 3. Indeed, poor clarity of emotions at Time 2 (*B* = 0.68, *SE* = 0.20, *p* = 0.001, 95% CI = [0.30, 1.07], *β* = 0.22) and difficulties with concentrating and accomplishing goals when experiencing negative emotions at Time 2 (*B* = 0.46, *SE* = 0.19, *p* = 0.017, 95% CI = [0.08, 0.84], *β* = 0.18) significantly and positively predicted boredom at Time 3 (path *b*), over and above participants’ BP, pre-existing stress, age, gender, pandemic trauma at Time 1, and emotion dysregulation at Time 2. Taken together, our theorized mediation model is supported even when BP is modeled and controlled for.

**Table 5:** Exploratory Mediation Analyses Controlling For Boredom Proneness (BP).

Mediator	*a* path			*b* path			*Indirect* Pathways to Boredom		
	*B (SE)*	*p*	*β*	*B (SE)*	*p*	*β*	*B (SE)*	*p*	*β*
Clarity	0.82 (0.24)	0.001	0.31	0.68 (0.20)	0.001	0.22	0.56 (0.23)	0.016	0.07
Goals	1.00 (0.22)	<0.001	0.32	0.46 (0.19)	0.017	0.18	0.46 (0.22)	0.034	0.06
Impulse	1.07 (0.25)	<0.001	0.39	−0.02 (0.24)	0.930	−0.01	−0.02 (0.26)	0.932	−0.00
Nonacceptance	0.82 (0.23)	<0.001	0.28	0.01 (0.20)	0.944	0.01	0.01 (0.17)	0.946	0.00
Strategies	1.00 (0.21)	<0.001	0.34	0.03 (0.21)	0.891	0.01	0.03 (0.21)	0.891	0.00

The *a* paths represent that path from pandemic trauma at Time 1 to emotion dysregulation at Time 2, while controlling for participants’ BP, pre-existing stress, age, and gender. The *b* paths represent that path from a facet of emotion dysregulation at Time 2 to boredom at Time 3, while controlling for participants’ BP, pandemic trauma at Time 1, other facets of emotion dysregulation at Time 2, pre-existing stress, age, and gender. BP significantly and positively predicted boredom at Time 3, over and above participants’ pandemic trauma at Time 1, emotion dysregulation at Time 2, pre-existing stress, age, and gender, *B* = 0.37, SE = 0.06, *p* < 0.001, 95% CI = [0.26, 0.48], *β* = 0.36.

## 4. Discussion

The COVID-19 pandemic, which has thrown the world into turmoil for more than 2 years, has underscored the pressing need to understand the effect of the pandemic on people’s emotional wellbeing, particularly in light of the increasing probability of experiencing future pandemics as severe as COVID-19 (e.g., Marani et al., 2021). Shifting away from the emphasis on external pandemic-related factors, such as constraint and monotony, as well as the emphasis on individual differences (e.g., BP), the current longitudinal study sought to examine (a) if trauma predicts subsequent boredom and (b) if emotion dysregulation mediates this relationship. The current study took place during the height of the pandemic, between 6 May and 18 June, 2020, where COVID-19 cases and deaths increased substantially and unemployment rates were at unprecedented levels in the U.S. This provided us with the timely opportunity to examine these emotional contributors of boredom, an aversive feeling associated with a host of psychosocial difficulties and risky behaviors that jeopardize the effectiveness of containment measures that are designed to protect people from the otherwise wide-ranging traumatic effects of pandemics (e.g., infection, exposure, income loss, and social isolation).

### 4.1. Overview and discussion of findings

Consistent with the above-reviewed research that links traumatic and stressful life experiences to the boredom (e.g., Stretch et al., 1996a,b; Silove et al., 1997; Chaney and Blalock, 2006; Pfefferbaum et al., 2007; Schweitzer et al., 2018; Volkert et al., 2013), people with more pandemic trauma at Time 1 and people a greater number of burdensome pre-existing (pre-pandemic) stressors felt more bored at Time 3, while controlling for all other variables in the hypothesized mediation model. Most notably, emotion dysregulation at Time 2 partially mediated the positive predictive relationship between pandemic trauma at Time 1 and boredom at Time 3, with medium indirect effects observed. In particular, people with greater pandemic trauma subsequently felt more bored when they lacked clarity on their emotions and had difficulties concentrating and accomplishing their goals in the face of negative emotions. Moreover, our exploratory mediation model revealed that poor emotional clarity and difficulties with engaging in goal-directed behaviors (fully) mediated the relationship between pandemic trauma at Time 1 and boredom at Time 3 when BP was included in the model, thereby highlighting the unique importance of these two facets of emotion regulation on people’s feelings of boredom during the pandemic. We discuss these facets of emotion regulation further.

As defined by the authors of the original and short-form versions of the DERS (Gratz and Roemer, 2004; Victor and Klonsky, 2016), emotional clarity taps into the extent to which people know (are clear about) the emotions they are experiencing. Emotional clarity is related to the concept of *emotional granularity*, which refers to individual differences in the specificity of one’s emotional experiences or a person’s ability to make fine-grained, nuanced distinctions between similar emotional states (see Barrett, 2004), as well as to the concept of *alexithymia*, which refers to dispositional difficulties with identifying and describing one’s emotions (see Sifneos, 1973). Indeed, the lack of clarity (as measured by the DERS), low emotional granularity, and alexithymia are linked with a host of psychosocial difficulties—as observed both before and during the COVID-19 pandemic: depressive symptoms, anxiety, stress, PTSD symptoms, emotional eating, and avoidance of unwanted thoughts/feelings, to name a few (e.g., Barrett, 2004; Gratz and Roemer, 2004; Hallion et al., 2018; van Strien, 2018; Tang et al., 2020). Compared to the other DERS subscales, the clarity subscale has a significantly stronger negative association with indices of emotional expressivity (i.e., the extent to which people outwardly display their emotions; Gratz and Roemer, 2004) and mindfulness (i.e., acting with awareness and describing one’s inner experiences; Lavender et al., 2016). Within the context of trauma, empirical work has underscored the role of complex or multiple adverse life experiences on people’s inability to differentiate their emotional experiences (see Eichhorn et al., 2014; Zorzella et al., 2020 for a review). One study published during the COVID-19 pandemic found that a higher number of exposures to coronavirus was positively associated with more severe alexithymia symptoms among home-quarantined adults, including difficulties identifying and difficulties describing one’s emotions (Tang et al., 2020). Within the context of boredom, several studies, as described earlier, suggest a positive link between difficulties with labeling and describing one’s emotions and BP (Seib and Vodanovich, 1998; Gana et al., 2000; Harris, 2000; von Gemmingen et al., 2003; Eastwood et al., 2007). However, these studies present cross-sectional and correlational results among trait-based measures of emotional clarity and boredom, which precludes the ability to make conclusions about the temporal relations and excludes an examination of boredom as a state. The current study not only shows that the lack of emotional clarity predicts greater feelings of boredom at a later time point, but that the lack of emotional clarity is a mechanism through which people who experience more pandemic trauma subsequently feel more bored. We posit that those with greater pandemic trauma are subsequently unable to understand, articulate, and express their feelings, which may leave them “adrift”—lacking clarity on what is important and valuable to them. Taking together the above-reviewed definition and psychodynamic and existential theories of boredom, as well as the above-reviewed literature on the DERS, the inability to identify what one feels prevents one from being able to express and articulate their desires, which is a critical precondition for becoming engaged—thus, giving rise to boredom.

The goals subscale of the DERS assesses people’s ability to concentrate, follow through, and accomplish goal-directed tasks when experiencing negative emotions. This conceptualization emphasizes the ability for a person to behave in accordance with their desired goals when experiencing negative emotions (Linehan, 1993; Melnick and Hinshaw, 2000; as cited in Gratz and Roemer, 2004). As with the clarity subscale, difficulties with engaging in goal-directed actions is linked to a range of psychological problems, such as depression, anxiety, and stress (e.g., Hallion et al., 2018), and some studies suggest that the goals subscale is more strongly related to mood and anxiety-related symptoms, obsessive–compulsive difficulties, interpersonal sensitivity, somatization, and paranoid ideation than majority of the other DERS subscales (e.g., Coutinho et al., 2010). Compared to the other DERS subscales, the goals subscale is more robustly and negatively associated with indices of negative mood regulation (i.e., the belief that some behavior or thought will alleviate a negative state or induce a positive one; Gratz and Roemer, 2004), as well as more robustly and positively associated with dysregulated behaviors, specifically disordered eating and partner abuse (Gratz and Roemer, 2004; Cooper et al., 2014). Like emotional clarity, the ability to engage in goal-directed or desired actions is hindered in times of stress and trauma. Experimental work has demonstrated that in stressful scenarios, people’s engagement in goal-directed behaviors declines and, rather, their engagement in habits or routine behaviors increases (Neal et al., 2013). Neuroscientific findings corroborate this work, showing that elevations of stress hormones reduce people’s ability to make decisions and plan, but instead, increase their engagement in established routines (Schwabe et al., 2012). As described in the introduction, studies have found that BP is related to trait-based indices of diminished self-regulation and goal pursuit (i.e., “state orientation” and “Assessment” regulatory mode; Blunt and Pychyl, 1998; Struk et al., 2015; Mugon et al., 2018). The current study builds upon this correlational work, demonstrating that difficulties with engaging in desired actions—in the face of pandemic traumatic stressors—predict subsequent feelings of boredom. Drawing together the above-reviewed theories and correlational work, we posit that people with more pandemic trauma subsequently feel more bored partially through difficulties with engaging in goal-directed actions because they are more preoccupied with intrusive and persevering thoughts about these stressors or are rigorously evaluating different possible response options and outcomes; this impairs their initiation and maintenance of goal pursuit, leaving them unengaged. Future work could examine if the relationship between people’s difficulties with engaging in goal-directed behaviors and subsequent boredom involves or is independent from their state orientation and Assessment mode of self-regulation.

### 4.2. Implications and future directions

The current longitudinal study underscores noteworthy points about people’s emotional functioning and boredom during the height of the COVID-19 pandemic—during a period of increasing COVID-19 cases, deaths, and widespread unemployment. First, the results of the current study elucidate the role of pandemic trauma—consisting of people’s exposure to coronavirus and the financial, resource-related, and interpersonal stressors of the pandemic—on emotion regulation. People with greater pandemic trauma subsequently struggled to understand and accept their emotions, engage in desired goal-directed behaviors, and control undesired impulsive behaviors, and they lacked the belief in their ability to cope. These findings have the potential to be important, as they highlight the *wide-ranging* impact of pandemic trauma on people’s ability to regulate negative feelings engendered by such trauma—thereby highlighting the need for transdiagnostic evidence-based supports that facilitate people’s ability to emotionally cope with the pandemic (e.g., Waterschoot et al., 2022). Second, the results of the current study advance our understanding of boredom by highlighting under-researched, but highly relevant, predictors of boredom during pandemics: trauma and emotion dysregulation. The longitudinal nature of the current study emphasizes that people’s trauma related to the pandemic, as well as people’s lack of clarity on their emotions and difficulties with pursuing their goals in the face of such emotions, are risk factors for feeling bored. Given the above-noted associations between boredom and risky rule-breaking behaviors during the pandemic, as well as the link between boredom and psychosocial dysfunction, a fuller understanding of the predictors of boredom is a critical first step to informing the development of boredom interventions that can help mitigate the deleterious impact of boredom on people’s wellbeing—both within and beyond the context of pandemics. The current study’s findings suggest that interventions that focus on enhancing people’s ability to identify and describe their emotions, as well as enhancing their engagement in actions that are aligned with their values and goals in the face of negative affect, may be important next steps in this regard. For example, Acceptance and Commitment Therapy, which teaches clients skills to notice, describe, and manage painful feelings and thoughts, as well as to clarify and pursue value-based goals (Harris, 2009), may be a viable avenue for boredom-specific interventions.

The current study is the first to longitudinally illustrate that trauma and emotion dysregulation predict people’s boredom during the pandemic, as well as that emotion dysregulation partially mediates the prediction of people’s pandemic trauma on their subsequent boredom. When people’s BP is accounted for, emotion dysregulation fully mediates the prediction of people’s pandemic trauma on their boredom. In addition to advancing our theory of boredom and informing potential boredom interventions, this novel understanding of boredom introduces the opportunity to ask additional questions about the emotional factors that contribute to boredom. First, the current study used a longitudinal approach that underscores the temporal relations between pandemic trauma, emotion dysregulation, and boredom during the pandemic; however, we acknowledge that without an experimental design, our ability to make firm causal conclusions about the relationships between these variables is limited. Second, our mediation model controlled for pre-existing stress, age, and gender, thus emphasizing that when these factors are held constant, people’s pandemic trauma and emotion dysregulation uniquely predict boredom. Future research could seek to explore the moderating role of these factors, thus elucidating if and how pre-existing stress, age, and gender enhance or diminish the impact of people’s pandemic trauma and emotion dysregulation on their boredom. In the current study, we controlled for pre-existing stress, an important marker of psychopathology, such as adjustment disorder. However, we did not measure and examine the impact of pre-existing psychopathology on the paths estimated in our hypothesized mediation model. It is possible that different results (i.e., enhanced or diminished effects of pandemic trauma and emotion dysregulation) may have emerged if we included pre-existing psychopathology in our model. Thus, future work that explores the potential effect of this variable (i.e., as a covariate and/or moderator) on the generalizability of our findings is encouraged. Third, our work introduces questions about the moderating role of dispositional traits in the pathways between pandemic trauma, emotion dysregulation, and boredom. For example, one trait pertinent to the current mediation model is experiential avoidance, defined as the unwillingness to remain in contact with uncomfortable private events (e.g., thoughts, emotions, sensations, memories, urges) by avoiding or escaping these experiences (Hayes et al., 1996). Although experiential avoidance is conceptually similar to both distress tolerance and emotion dysregulation, evidence suggests that experiential avoidance is distinct from both of these constructs (for a review, see Boulanger et al., 2010) and predicts psychopathology above and beyond general psychological distress (e.g., Gratz et al., 2008). In keeping with this literature, it is plausible that pandemic trauma (at Time 1) may subsequently predict greater emotion dysregulation (at Time 2) among people who are more unwilling to remain in contact with their inner experiences. Fourth, the current study introduces the possibility that other relevant constructs may mediate the predictive relationship between pandemic trauma and boredom. Traumatic and adverse life events not only result in emotion dysregulation, but these experiences also shake one’s meaning in life, which otherwise help people cope (e.g., Larner and Blow, 2011) and buffer against the effects of stressful life experiences on emotional functioning (e.g., Halama, 2014). Longitudinal work suggests that life meaning predicts lower boredom propensity across time, and experimental work suggests that manipulating perceptions of life meaning significantly changes boredom, such that people with low life meaning feel significantly more bored than those with high life meaning (Fahlman et al., 2009). As Van Tilburg and Igou (2012) have showed, boredom is differentiated from anger, frustration, and sadness insofar as involves a perception that one’s situation is meaningless (see also van Tilburg and Igou, 2017; Chan et al., 2018). Thus, future directions might consider examining if disrupted meaning is an additional mechanism through which pandemic trauma predicts boredom. Finally, additional longer-term longitudinal studies are needed to understand the nature of the relationships between trauma, emotion dysregulation, and boredom beyond the time points in this study. These studies will be especially critical when examining long-term and residual effects of the pandemic.

## 5. Conclusion

Prior research suggests that external constraints and monotony imposed by the pandemic and individual differences in BP are contributors to boredom during the pandemic. The current longitudinal study builds on this important work, finding that trauma and difficulties with regulating emotions are contributors to boredom during the pandemic. People with greater pandemic trauma subsequently feel more bored when they lack clarity on their emotions and struggle to engage in goal-directed behaviors when experiencing negative emotions. These findings provide another way of understanding why boredom is prevalent during a pandemic. These findings also underscore specific areas of emotional functioning (i.e., emotional clarity and engagement in goals) that can be targeted to help reduce boredom during this and other taxing and stressful times, and, relatedly, to help mitigate the psychosocial difficulties associated with boredom and boredom-related non-adherence to critical lifesaving public health measures.

## Data availability statement

The raw data supporting the conclusions of this article will be made available by the authors, without undue reservation.

## Ethics statement

The studies involving human participants were reviewed and approved by York University Human Participants Review Committee (Certificate #: e2020-119). The patients/participants provided their written informed consent to participate in this study.

## Author contributions

VB, AW, and JE: conceptualization and writing—review and editing. VB: data analysis and writing—original draft. JE: supervision. All authors contributed to the article and approved the submitted version.

## Funding

This research was funded by grants to JE from the Natural Sciences and Engineering Research Council of Canada (NSERC; Grant #: 496399).

## Conflict of interest

The authors declare that the research was conducted in the absence of any commercial or financial relationships that could be construed as a potential conflict of interest.

## Publisher’s note

All claims expressed in this article are solely those of the authors and do not necessarily represent those of their affiliated organizations, or those of the publisher, the editors and the reviewers. Any product that may be evaluated in this article, or claim that may be made by its manufacturer, is not guaranteed or endorsed by the publisher.

## Footnotes

^1^ https://osf.io/8pexf/

^2^There were baseline (i.e., Time 1) differences between participants who completed all three time points (*N* = 345) and thus were included in the hypothesized mediation model and participants who did not complete all three times points (i.e., they completed only Time 1 or Time 1 and 2; *N* = 1,011). The latter group endorsed higher scores on three of the four pandemic trauma indicator variables (excluding resource-related stress), the five DERS-18 subscales, and the MSBS, as well as reported a greater number of burdensome pre-existing stressors and were younger in age (all *p*’s < 0.05). That the latter group struggled more with pandemic stressors and emotion dysregulation at the start of the study, as well as reported more burdensome pre-existing stressors, could be why they did not continue to participate after Time 1 or Time 2 in light of such taxing and challenging circumstances.

^3^This exploratory mediation model was estimated during the review of this manuscript. We thank the Reviewers of this manuscript for their suggestion.
